# The Role of the Multiparametric MRI LiverMultiScan^TM^ in the Quantitative Assessment of the Liver and Its Predicted Clinical Applications in Patients Undergoing Major Hepatic Resection for Colorectal Liver Metastasis

**DOI:** 10.3390/cancers15194863

**Published:** 2023-10-05

**Authors:** Tarak Chouari, Nabeel Merali, Francesca La Costa, Jonas Santol, Shelley Chapman, Alex Horton, Somaiah Aroori, John Connell, Timothy A. Rockall, Damian Mole, Patrick Starlinger, Fenella Welsh, Myrddin Rees, Adam E. Frampton

**Affiliations:** 1 MATTU, The Leggett Building, Daphne Jackson Road, Guildford GU2 7WG, UK; t.chouari@nhs.net (T.C.);; 2 Department of Hepato-Pancreato-Biliary (HPB) Surgery, Royal Surrey County Hospital, Egerton Road, Guildford GU2 7XX, UK; 3 Oncology Section, Department of Clinical and Experimental Medicine, Faculty of Health and Medical Science, University of Surrey, Guildford GU2 7WG, UK; 4 Department of Surgery, HPB Center, Vienna Health Network, Clinic Favoriten and Sigmund Freud Private University, 1090 Vienna, Austria; 5 Institute of Vascular Biology and Thrombosis Research, Center of Physiology and Pharmacology, Medical University of Vienna, 1090 Vienna, Austria; 6 Department of Radiology, Royal Surrey County Hospital, Egerton Road, Guildford GU2 7XX, UK; 7 Department of Surgery, Division of Hepatobiliary and Pancreatic Surgery and Transplant Surgery, Derriford Hospital, Plymouth PL6 8DH, UK; 8 Perspectum Ltd., Oxford OX4 2LL, UK; 9 Clinical Surgery, Royal Infirmary of Edinburgh, University of Edinburgh, Edinburgh EH10 5HF, UK; 10 Centre for Inflammation Research, University of Edinburgh, Queen’s Medical Research Institute, Edinburgh EH105HF, UK; 11 Department of Surgery, Division of Hepatobiliary and Pancreatic Surgery, Mayo Clinic, Rochester, MN 55902, USA; 12 Center of Physiology and Pharmacology, Medical University of Vienna, 1090 Vienna, Austria; 13 Department of Surgery, Medical University of Vienna, General Hospital, 1090 Vienna, Austria; 14 Hepato-Biliary Unit, Hampshire Hospitals Foundation Trust, Basingstoke, Hampshire RG24 9NA, UK

**Keywords:** colorectal liver metastasis, post-hepatectomy liver failure, prehabilitation, hepatectomy, LiverMultiScan, magnetic resonance imaging, quality of future liver remnant

## Abstract

**Simple Summary:**

This narrative review summarises the current limited literature relating to multiparametric magnetic resonance imaging software (called LiverMultiScan^TM^) used for the purpose of analysing liver tissue in a non-invasive approach to assess liver health. Liver health is of particular importance when considering patients with liver malignancy planned for a major liver resection. The aim of this review is to consider the current evidence for its use in the setting of chronic liver disease, liver malignancy, and perioperative planning, and to examine the future applications of such software and the hurdles it must surpass to improve patient selection and outcomes in liver surgery.

**Abstract:**

Liver biopsy remains the gold standard for the histological assessment of the liver. With clear disadvantages and the rise in the incidences of liver disease, the role of neoadjuvant chemotherapy in colorectal liver metastasis (CRLM) and an explosion of surgical management options available, non-invasive serological and imaging markers of liver histopathology have never been more pertinent in order to assess liver health and stratify patients considered for surgical intervention. Liver MRI is a leading modality in the assessment of hepatic malignancy. Recent technological advancements in multiparametric MRI software such as the LiverMultiScan^TM^ offers an attractive non-invasive assay of anatomy and histopathology in the pre-operative setting, especially in the context of CRLM. This narrative review examines the evidence for the LiverMultiScan^TM^ in the assessment of hepatic fibrosis, steatosis/steatohepatitis, and potential applications for chemotherapy-associated hepatic changes. We postulate its future role and the hurdles it must surpass in order to be implemented in the pre-operative management of patients undergoing hepatic resection for colorectal liver metastasis. Such a role likely extends to other hepatic malignancies planned for resection.

## 1. Introduction

Liver biopsy remains the historical “gold standard” for measuring fibrosis, steatosis, and steatohepatitis, despite being both invasive and costly, as well as sampling-dependent, taking only a snapshot (0.002%) of liver tissue, and observer-dependent with associated risks that limit patient acceptability [1,2,3,4,5]. However, with an epidemic of progressive liver disease, in combination with the implications of chemotherapy on liver health, the importance of adequately stratifying liver disease severity has never been more pertinent. There is a need for non-invasive, reliable, and objective methods to assess liver histopathology of the functional liver remnant (FLR) prior to major hepatic resection. Such novel tests may stratify disease severity, identify patients at risk of liver decompensation, and inform clinical decision making without the limitations associated with liver biopsy in the context of major hepatic resection.

Imaging offers an attractive, non-invasive method, which may address the unmet need highlighted above. Imaging provides an anatomical assessment of the liver, and recent work suggests it may have a role in the histological assessment of the liver in either a focused or entire liver approach, thereby eliminating the limitations described above with the added value of amalgamating assessments of specific anatomical areas of interest with pre-operative planning. Specifically, magnetic resonance imaging (MRI) is already a leading modality in the assessment of colorectal liver metastases (CRLM) and is commonly used. Therefore, any imaging biomarkers utilising MRI would be of great value in this field, without any additional steps required in the pre-operative workup and minimising further inconveniences to the patient. Non-invasive MR imaging markers of histopathological features of the liver warrant precise assessment in the pre-operative assessment of CRLM and have associations with long-term outcomes in other liver diseases [6,7,8,9]. In this review, we discuss the recent technological advances and evidence for one specific multiparametric MRI software, the LiverMultiScan^TM^ (Perspectum, Oxford, UK). As a recent novel technology, there is limited evidence focusing solely on its role in hepatic surgery for CRLM. Thus, we will discuss its demonstrated application in chronic liver disease, as well as hepatic surgery. We foresee this technology as having a potential role in various aspects of hepatic surgery and postulate potential roles it may serve, as well as hurdles it must surpass to be implemented in the pre-operative management of patients undergoing liver resection for CRLM and other hepatic malignancies.

## 2. The LiverMultiScan^TM^

The LiverMultiScan^TM^ uses information from a novel multiparametric MR and PDFF protocol that allows for the in vivo objective characterisation of liver tissue. The software processes T1 mapping of extracellular water content, which is a proxy for inflammation/fibrosis, T2* mapping for liver iron content and proton density fat fraction (PDFF) for liver fat quantification. However, T1 measurements are adjusted for the iron level, as high levels of iron can lead to decreased T1 values or pseudo-normal values, thus providing a corrected T1 value (cT1) [10,11]. Banerjee and colleagues conducted a prospective, comparative, non-randomised study comparing LiverMultiScan^TM^ with histological assessment of tissue obtained from liver biopsy in an unselected cohort [10]. They showed a good correlation of the LiverMultiScan^TM^ with histological parameters in a cohort with an array of liver disease aetiologies. For discriminating any healthy individuals with any degree of fibrosis, steatosis, or iron content, they showed an area under the curve (AUROC) of 0.94 (*p* < 0.0001, 95% CI 0.90–0.99), 0.93 (95% CI 0.87–0.99, *p* < 0.0001), and 0.94 (95% CI 0.87–1.00, *p* < 0.0001), respectively. However, it is unclear if any of the patients in this study had operable primary or secondary hepatic malignancies. 

**Figure 1:**
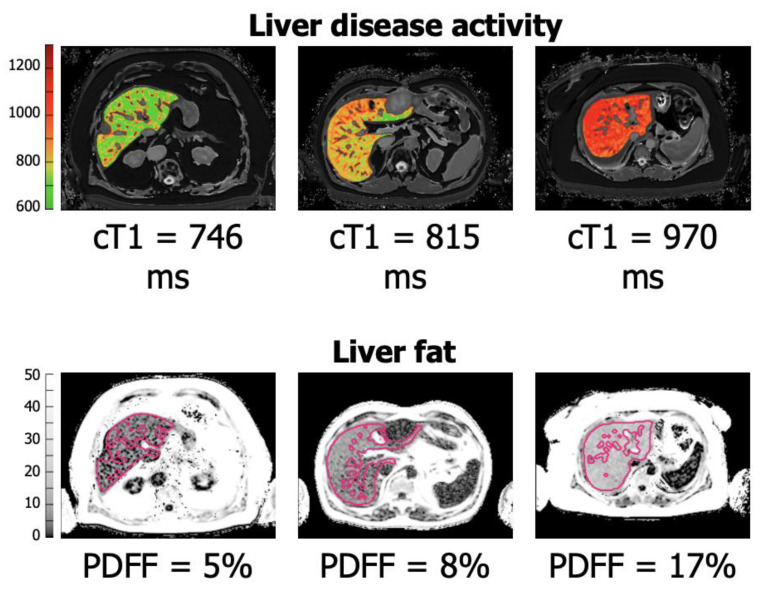
LiverMultiScan^TM^ schematic demonstration of the increasing degrees of liver disease activity/fibrosis (as measured by cT1 score) and liver fat content (as measured using PDFF) in 3 patients included in the Precision1 trial. Scores are graphically represented on an increasing colour scale on the liver. Images reproduced with permission from the Basingstoke Unit and Perspectum ltd.

In terms of practicality, there is no additional hardware required to carry out the scans. The only requirement is appropriate software, which is suitable for most modern MRI scanners. Furthermore, there is no specific training required for the clinician to implement this technology. However, clinicians should have a level of education on how to correctly interpret the clinical results. Only the MRI technicians/radiographers require specific training to acquire the scans correctly, which takes approximately 2 h to complete. The analysis of data obtained does not require the involvement of a radiologist. In fact, the images/data obtained are securely uploaded (by a radiographer) to the manufacturer (Perspectum Ltd., Oxford, UK). Once uploaded, the data are analysed by their team using their propriety software. The results were previously corroborated in an independent study conducted by the Hepatobiliary teams at the University of Edinburgh and Basingstoke Hospital [12]. A report (quantifying cT1, T2*, and PDFF) with maps of the liver is returned to the clinical team. The overall analysis has a turnaround time of approximately 2 h. Figure 1 illustrates the different degrees of parenchymal wellbeing in terms of cT1 and PDFF.

There are currently over 600 MRI scanners with the technology, and there is regulatory clearance for the clinical use of the technology in the USA, Singapore and across Europe (including the United Kingdom). It should be noted that LiverMultiScan adds an additional 15 min to a standard MRI Liver scan. 

In terms of cost effectiveness, no study to date has explored the cost effectiveness of implementing the LiverMultiScan^TM^ for the purpose of CRLM or other hepatic cancers. However, it was deemed a cost-effective alternative to liver biopsy in the National Health Service (NHS) for monitoring autoimmune hepatitis [13] and for the risk stratification of NAFLD [14]. 

### 2.1. LiverMultiScan ^TM^: The Grading of Hepatic Fibrosis and Future Applications in Hepatic Surgery

Research to date suggests that liver parenchyma T1 relaxation time (milliseconds) maps of the liver provide information related to tissue composition, as T1 relaxation time increases with fibrosis and thus provides a measurable parameter used in the quantification of liver fibrosis in chronic liver disease [10,15,16,17,18]. Previous work also suggests cT1 mapping may be able to distinguish between mild (Ishak 1–2) and severe (Ishak 5–6), as well as between moderate (Ishak 3–4) and severe disease, but not between mild and moderate grades of histological fibrosis; however, its true accuracy in doing so remains to be determined [10,16,19,20]. If LiverMultiScan^TM^ cT1 scores cannot differentiate between lower grades of fibrosis, its value may be limited in high-stakes complex surgery, which relies on detail and marginal gains. Some have investigated the relationship between grades of fibrosis and outcomes in disease states where hepatic surgery is indicated. Some work is described below and provides insight into how we may utilise the LiverMultiScan^TM^ to predict outcomes prior to embarking on surgical intervention. 

One group showed that the fibrosis grades of Ishak 1–5 have no correlation with survival outcomes after liver resection for Hepatitis B-associated Hepatocellular Carcinoma [21]. However, Ishak 6 (i.e., Cirrhosis) was independently associated with poor overall recurrence-free survival and overall survival [22]. This study did not look at intraoperative or postoperative outcomes other than survival and recurrence. One limitation is that the histological grading was based on the resected sample as opposed to the remnant. Other work has not shown any difference between patients with varying grades of fibrosis and intraoperative outcomes nor immediate postoperative complications following right major hepatectomy for HCC [23]. However, an association between the length of stay and fibrosis grade was noted. Furthermore, this group demonstrated that rates of remnant liver volume growth at 6 and 12 months following right major hepatectomy are inversely associated with the severity of fibrosis. Whilst some work corroborates their findings [24,25], others contradict this statement [26]. In CRLM, high levels of histological fibrosis may be related to hepatic-specific recurrence-free survival [27]. The retrospective nature, lack of power, and lack of standardised histological assessment found in all these studies limit their interpretation and warrant further validation. Indeed, it is a major challenge to obtain reliable and standardised liver histological grading. cT1 scores may provide such standardisation. 

Whilst it is logical that chronic liver disease and fibrosis affect Post-Hepatectomy Liver Failure (PHLF) and other outcomes such as RFS and OS (especially in HCC), the nuance of fibrosis grades and its association with outcomes after liver resection for CRLM and other hepatic malignancies remains to be determined. This should be a focus of future work in order to guide decision making in a field where the role of imaging markers is likely to only gain traction and relevance. Imaging biomarkers for grades of fibrosis may indeed provide valuable insights into the outcomes of hepatic surgery and may prove to be a key prognostic marker. 

There are other considerations for the T1 measurement. Importantly, T1 or cT1 are indirect measures of liver fibrosis and may be prone to confounding factors. The T1 mapping technique may be affected by fat levels, whereby it is shorter in regions of fat [28,29]. However, cT1 was also shown to correlate with the histological grade of steatohepatitis [30] and correlates well between no fibrosis and fibrosis of any grade in the presence of steatohepatitis, albeit at higher cut-off values for cT1 [10]. Thus, the true T1 value may well be the weighted sum of hepatic fat content, liver fibrosis/inflammation, and any other factors which may affect T1 measurements. T1 is also affected by inflammation [31], which is logical when we consider that extracellular water content rises with chronic fibrosis and acute inflammation [11]. This may raise questions regarding the clinical applicability of cT1 alone in the presence of acute inflammation, hypoalbumenia/fluid overloaded status, altered lipid content, and/or longstanding liver disease following chemotherapeutic intervention for hepatic malignancies or following any form of pre-habilitation dietary-based intervention, preoperative sarcopenia, and postoperative nutritional support. It is likely that one would have altered cT1 scores, which may result in the misinterpretation of fibrosis and thus, validation tests must be conducted to establish cT1 cut-offs for various grades of fibrosis in the context of underlying patient factors. An urgent study into the nuances of the relationships between these patient factors and cT1 is warranted in order to correct the cT1 score further and improve the interpretation of cT1, especially when we consider that the optimisation of imaging parameters was shown to improve the impact of fat on cT1 [28]. cT1 correlation with histological disease features is maintained even after controlling for steatosis [32]. cT1 is also affected by patients with type 2 diabetes, resulting in possibly higher values [29]. Interestingly, cT1 mapping is unaffected by the presence of ascites, which is important to note in the context of intra-abdominal malignancy [10].

With the LiverMultiScan^TM^ software, it could be possible to demonstrate which parts of the liver are spared/unaffected by unhealthy parenchyma, and one can highlight regions of interest. Thus, it may provide further insights into the state of the future liver remnant. However, we must be sure that we do not include fluid-filled structures, such as the porta hepatis/large vessels, which theoretically could cause interference. Thus, local expertise or outsourcing expertise is paramount to future implementation. Using the software developed by Banerjee and colleagues, a cT1 timing map can be converted into a schematic representation of the liver whereby increasing fibrosis (i.e., increasing cT1 time) is graphically represented on an increasing colour scale on the liver, which again may have its uses in the pre-operative planning of liver resection, as well as in both patient and surgical team education and understanding. In fact, one study demonstrated that providing patients (with an array of liver diseases) with a LiverMultiScan^TM^-based visual report improved patient comprehension and experience [33]. This is, of course, imperative in oncological surgery. One possible limitation is that, naturally, one will find discrepancies between the planned and actual resection planes.

Eddowes and colleagues carried out an independent validation study to assess the diagnostic accuracy of the LiverMultiScan^TM^ in predicting the severity of NAFLD [19]. cT1 had an excellent ability to identify patients with any fibrosis compared to healthy controls (AUROC 0.93, 0.86–1.00). However, this study found only a moderate association with the steatosis or fibrosis stage in their cohort when comparing low-risk patients (simple steatosis and less than or equal to F1 grade fibrosis) vs. high-risk patients (Patients with NASH or >F1 fibrosis) with AUROC 0.73 (0.53–0.93). Whilst contradictory to previous work [10,34], this study had a small sample size and poorly matched patient groups with regard to cohort size (50 vs. 6 patients) and demographics. Furthermore, they used an alternative fibrosis grading system compared to previous studies [10,30,34]. It is likely that the variable results between studies may be attributable to the factors listed above, as well as the fact that studies are comparing the intervention with a proposed ‘gold standard’ biopsy, which, in fact, has limitations such as significant inter-observer variability and a limited sample of the liver, which may not be representative of any future liver remnant. Large multi-centre clinical studies in disease-specific cohorts will hopefully shed further light on the discrepancies found between these studies and will likely pick up more subtle relationships between cT1, histological fibrosis grading, and clinical outcomes. Comparing the LiverMultiScan^TM^ grading of fibrosis with a larger sample of tissue (for example, a sample taken during hepatectomy) may produce more meaningful comparisons in the future. 

Taking all of this into account, Eddowes et al. showed a sensitivity of 88% and specificity of 100% for the detection of NAFLD using multiparametric MRI [19]. They went on to investigate other non-invasive tools for predicting the degree of fibrosis and found other markers to be superior to cT1. Their work correlates with other studies that have also shown the value of other non-invasive tests in predicting the grade of fibrosis [35,36,37]. Pavlides and colleagues used cT1 mapping in patients with chronic liver disease to show a correlation between the degree of disease and the risk of developing clinical events (ascites, encephalopathy, mortality, and HCC) with 100% negative predictive value [34]. T1 mapping was also shown to differentiate Child–Pugh A patients from Child–Pugh B or C effectively (0.00001) [38]. cT1 scores were also reported to correlate with portal hypertension [39,40], which, of course, correlates with PHLF [41]. Such additional data may provide crucial information in order to guide appropriate treatment modalities or adjuncts, which may improve any future liver remnant.

The role of the LiverMultiScan^TM^ assessment of fibrosis and predicting clinical outcomes in chronic liver disease was previously demonstrated in a number of other studies as well [15,34,42,43]. However, the role of multiparametric MRI in predicting outcomes in patients undergoing resection largely remains to be determined and should be assessed in future studies. It is logical to consider the importance that a non-invasive assay of the histological makeup of the liver remnant will have on predicting outcomes in liver surgery. In fact, Mole et al. alluded to this [12]. In a multicentre observational clinical trial, this group used cT1, PDFF, and T2 mapping overlaid onto an estimated 3D image of a future liver remnant model created for an unselected group of patients undergoing liver resection to characterise the liver tissue of the future liver remnant. They showed that for patients where more than 10% of the liver volume was removed (*n* = 77), the median length of stay post-liver resection was longer in patients who had a cT1 score above the upper limit of normal compared to those with a cT1 score below this level (Wilcoxon rank sum test, *p* = 0.0053). Furthermore, they showed that a preoperative cT1 score above the upper limit of normal is associated with a higher Hyder–Pawlik score (a weighted score of bilirubin, INR, and creatinine), suggesting that cT1 may correlate with scoring systems aimed at predicting morbidity. A composite score of future liver remnant volume and cT1 showed reasonable diagnostic accuracy in discriminating patients with a high 5-day sum of modified Hyder–Pawlik scores in the upper quartile, with an AUROC of 0.78 (95% CI 0.66–0.90). This composite score performed better than FLR volume alone (AUROC 0.70 (95% CI 0.55–0.84). Most patients in this study had CRLM (114/135 participants, 84%), and the median future liver remnant size was 83%. The main limitation of this study is that there were a limited number of patients who developed PHLF, and thus, the power in measuring the intended outcome is low. Furthermore, this study incorporated a modified Hyder–Pawlik score, which was not validated as a clinical tool. However, it utilises serum measures of liver dysfunction, which are commonly used to monitor patients in the post-hepatectomy period and are already used in the original Hyder–Pawlik scoring system. A study into the clinical validity of the modified Hyder–Pawlik scoring system should be considered to further our understanding of the clinical utility of the cT1’s role in predicting morbidity following liver resection. Furthermore, large independent multi-centre studies directly comparing cT1, as well as measures of liver fat with subsequent morbidity/mortality post hepatectomy may be of great benefit. 

Furthermore, this study compared patients with any degree of fibrosis vs. no fibrosis, i.e., cT1 score at the upper limit of normal. The benefit of the non-invasive assessment of hepatic fibrosis in patients undergoing hepatectomy may lie in patients who are borderline with regard to whether they would or would not be suitable for resection due to the risk of PHLF. Therefore, the benefit of such an assay may not lie in comparing patients with any degree of fibrosis and no fibrosis but, in fact, delineating between specific grades of fibrosis.

### 2.2. Alternative Imaging Markers of Liver Fibrosis and Fibrosis Grading

Previously, transient elastography (TE) and magnetic resonance elastography (MRE) gathered interest due to their ability to correlate well with the liver fibrosis stage and disease progression [16,19,44,45,46,47]. The main disadvantage of TE/MRE is that both require additional hardware, which might limit implementation, as well as specific software to acquire and process the information, whilst LiverMultiScan^TM^ can be used on any modern clinical 1.5 or 3.0 Tesla MR scanner with no additional hardware required. MRE/TE results may be confounded by acute inflammation, changes in transaminases, and cholestasis [48,49,50,51,52]. Furthermore, elastography has limited diagnostic use in iron overload, obese populations, and those with ascites [47]. Its role in quantifying liver health in the context of malignant liver disease planned for resection may be limited. TE specifically has a high failure rate of 18.4%, and cut-off values of liver stiffness for the different stages of liver fibrosis are not well established [48,53]. It was shown that the technical success rate of TE is 85% compared to 98.1% with the LiverMultiScan^TM^ for measuring fibrosis [44,49]. Furthermore, the longitudinal assessment of fibrosis using elastography is limited by operator and patient factors, which result in significant variations in measurements, affecting reproducibility. Therefore, its application in the serial assessment of the liver in the context of clinical studies or preoperative assessment may prove inferior to alternative tools [16,54]. On the other hand, cT1 measurement was shown to have low measurement failure rates, high repeatability, and reproducibility that are superior to elastography techniques [16,55,56,57]. McDonald and colleagues in 2018 carried out a prospective two-centre validation study assessing LiverMultiScan^TM^’s ability to measure hepatic inflammation, fibrosis, fat, and iron load compared to liver biopsy and compared it with transient elastography in unselected patients undergoing a liver biopsy for the investigation of chronic liver disease. They found the cT1 measurement of hepatic fibrosis/inflammation to be positively associated with liver biopsy (*p* < 0.001). They found no significant difference between the accuracy of the two tests in detecting any degree of histological change compared to normal subjects in an unselected population. TE was, however, superior with regard to identifying those with moderate–severe fibrosis and severe fibrosis. However, in subgroup analysis, where post-liver transplant patients were removed from the cohort, cT1 showed superior predictive accuracy in differentiating between groups of no fibrosis vs. any grade of fibrosis. Although transient elastography is available within the National Health Service (NHS), it is unlikely to serve as a tool for serial investigations of the liver, as it may not track changes in the liver reliably. The main benefit of LiverMultiScan^TM^ over MRI elastography is the lack of need for additional hardware, as well as the patented iron correcting T1 measurement. However, unlike cT1, MRE was shown to correlate well with all specific grades of histological fibrosis as measured by Ishak grading [58].

### 2.3. LiverMultiScan^TM^: Grading of Hepatic Steatosis/Steatohepatitis and Applications in Hepatic Surgery

It is clear that steatosis and steatohepatitis measures may also be of value in the preoperative assessment of patients undergoing liver resection, with a possible role in predicting intraoperative and postoperative outcomes [59,60,61,62,63,64]. LiverMultiScan^TM^ was initially used with MR Spectroscopy (MRS) or, more recently, proton density fat fraction (PDFF) for liver fat quantification. Banerjee et al. showed that MRS thresholds of 1.5% and 7.5% of the water signal could discriminate between different grades of steatosis, as measured by Brunt grading [10]. This study also showed that fat is homogeneously distributed throughout the liver, suggesting that one can use a resected liver tissue sample as a surrogate marker for the fat content of the liver remnant. The measurement of hepatic lipid content was previously described using several methods. However, spectroscopy was shown to be superior to others and is considered the optimum non-invasive measure of liver fat with regard to reliability, reproducibility, and validation as an accurate tool in measuring liver fat [10,65,66,67,68,69,70,71,72,73,74]. Recently, MRI-PDFF has shown its utility for liver fat assessment [74]. A recent meta-analysis showed that MRI-PDFF has excellent linearity and negligible bias with respect to MRS measurements over the entire range of steatosis severity [74]. Several pertinent studies are discussed below.

MRI-PDFF has been increasingly used to estimate fat infiltration and was shown to have high predictive accuracy for individual steatosis grades (Brunt grading) with AUROC ranging from 0.90 to 0.94 in unselected populations [16] and can detect changes in hepatic fat as small as 1% [75,76,77]. It was also shown to highly correlate with MRS [74] and hepatic triglyceride levels [75,76]. MRI-PDFF and MRS are reproducible [75].

A single-centre prospective clinical trial investigated whether a multiparametric 3D-MRE protocol combined with MRI-PDFF can be used to monitor histological improvements in NASH in 40 patients who underwent bariatric surgery for steatohepatitis. MRI-PDFF was compared to histological sampling taken intra-operatively and at 1 year follow-up, demonstrating changes in MRI-PDFF are associated with changes in histological steatosis and overall NAFLD activity score improvement after intervention. They suggest that MRI-PDFF is an ideal candidate for the longitudinal monitoring of non-alcoholic steatohepatitis after pharmacological therapy or lifestyle changes [78]. Indeed, the application of cT1 and MRI-PDFF are gaining popularity; a recent randomised control study investigated the role of treatments aimed at reducing hepatic fibrosis using cT1, as well as MRI-PDFF as a marker for changes in liver fibrosis and fat content [57]. It should be noted that MRI-PDFF measurements of fat are not affected by the presence of inflammation or fibrosis [77]. It is likely that MRI-PDFF correlates with steatosis severity in patients with CRLM. One study showed the preoperative measure of liver fat % shown on MRI-PDFF correlates in a stepwise fashion with increasing grades of steatosis assessed histologically on resected liver tissue in an unselected population undergoing liver resections for a range of malignancies of whom the majority of participants had CRLM [12]. 

Recently, clinical trials in liver surgery have alluded to the benefit a preoperative low-fat liver diet may have in patients planned for hepatectomy. Barth et al. performed a multi-centre randomised controlled trial on the effects of a one-week (low in calorie and fat) diet in patients undergoing liver surgery. Results showed a significant difference in easier manipulation of the liver and a decrease in operative blood loss [79]. We foresee novel imaging biomarkers having a quantitative role in the serial assessment of such dietary interventions and in establishing which patients would benefit from interventions aimed at decreasing liver fat content. Specifically, we wonder whether diet modification in the prehabilitation period has any implication on outcomes in liver resection and whether MR spectroscopy PDFF can be used to identify which patients would benefit most from interventions aimed at decreasing hepatic fat content and what degree of change is associated with positive outcomes in liver resection. Proving the reversibility of established steatosis and non-alcoholic steatohepatitis with preoperative intervention is key to interventions aimed at these concepts. However, it is probable that LiverMultiScan^TM^ (or alternative non-invasive imaging biomarkers) will serve a future purpose in both the research and clinical setting related to pre-operative fat modification. 

### 2.4. Future Clinical Applications of the LiverMultiScan^TM^ in Neoadjuvant Chemotherapy Setting in CRLM and Decision Making Regarding Optimum Treatment Modalities

Another exciting prospect is the role of multiparametric MRI in quantifying and predicting the effect neoadjuvant chemotherapy regimens have on liver health and subsequent outcomes, especially in the context of CRLM. Only 20% of patients with CRLM have initially resectable disease [80,81]. Neoadjuvant/downgrading chemotherapy prior to curative liver resection was shown to downsize the tumour burden and increase the number of patients suitable for resection [82,83]. Neoadjuvant therapy was shown to improve progression-free survival in patients with primary resectable CRLM [82,84]. Naturally, more patients are being considered for chemotherapy prior to surgical intervention. However, there are concerns regarding the administration of several cytotoxic agents and pathological liver changes referred to as chemotherapy-associated liver injury (CALI) [85,86,87]. One study demonstrated that CALI may increase the risk of PHLF by 11% [64]. Fluorouracil (5-FU) administration increases hepatic steatosis [59,88]. However, to date, the post-operative association of 5-FU-induced hepatic steatosis may be limited to increased infection rates [89] and bilirubin counts [90]. Irinotecan use in CRLM is associated with chemotherapy-associated steatohepatitis (CASH) [63,86,88,90,91]. CASH associated with irinotecan was shown to increase morbidity and mortality after partial hepatectomy for CRLM [63,64,91]. In fact, one study showed it is associated with a higher 90-day post-operative mortality (14.7%) compared to patients without evidence of CASH secondary to irinotecan (1.6%) [63]. Oxaliplatin may induce fibrosis [88,92,93]. The specific liver injury effect of oxaliplatin is called sinusoidal obstruction syndrome (SOS); it carries a 2.2-fold increase in the risk of developing it after oxaplatin-based therapies [86], and it is histologically characterised by hepatic sinusoidal dilatation, hepatocyte atrophy, perisinusoidal fibrosis, and nodular regenerative hyperplasia [87]. Studies showed that oxaliplatin-associated SOS increases the risk for post-hepatectomy morbidity as well [63,64,94,95]. Furthermore, the incidence of steatosis in patients undergoing chemotherapy for CRLM is between 30 and 47% [59,60]. With neoadjuvant chemotherapy coming to the forefront of the management of CRLM, the accurate preoperative evaluation and diagnosis of histological changes associated with CALI are of great importance. It is also worth noting that SOS undergoes a segmental development, which may limit the applicability of biopsy, and it is in this void that imaging biomarkers may serve useful, as would serological markers.

Welsh et al. were one of the first groups to highlight the risk of morbidity following prolonged neoadjuvant chemotherapy on liver outcomes [96]. In the same study, this group demonstrated a significant reduction in surgical complications with increasing time intervals between the cessation of chemotherapy and hepatic resection. These findings were not correlated with liver histological changes post-chemotherapy. One study showed that morbidity after liver resection is related to the number of neoadjuvant chemotherapy cycles, whereby greater than or equal to six cycles was associated with increased morbidity (54% vs. 19%, *n* = 45, *p* < 0.05) as well as six or more cycles being predictive of SOS (risk ratio = 3.198;95% CI [1.010–10.128] *n* = 90, *p* < 0.05) [64]. Other studies showed that nine or more cycles are an independent risk factor for PHLF [97]. Such evidence suggests there may be a close correlation between post-operative outcomes and the timing of surgical intervention post chemotherapy, as well as the number of neoadjuvant chemotherapy cycles, which may have implications on CALI and post operative outcomes. The non-invasive histological assessment of liver parenchyma post chemotherapy and prior to hepatectomy may serve as an important tool in the workup and decision making in patients with CRLM in the neoadjuvant setting.

What we need to understand is how does any specific chemotherapy regimen affect the liver of an individual patient? Can we predict which patients will be most affected by neoadjuvant chemotherapy and what specific degree of histological changes post-chemotherapy correlates with outcomes following hepatectomy? Does the LiverMultiScan^TM^ software correlate with grades of SOS, CALI, and CASH? If this is demonstrated and validated in future work, such non-invasive assessments of the liver prior to chemotherapy may help select specific chemotherapy regimens best suited to any given patient based on their parenchymal characteristics and predisposition to any given cytotoxic agent. It may also provide a degree of real-time surveillance of the hepatic parenchyma during neoadjuvant chemotherapy or after in the pre-operative period to guide optimum time for surgical intervention. Furthermore, longitudinal assessments of the liver during chemotherapy regimens may serve to ensure risk-benefit is addressed throughout treatment, ensuring an adequate response is balanced with optimised liver health. Non-invasive hepatic histological markers applications could be extended to prehabilitation strategies aimed at modulating the liver during or after chemotherapy and prior to resection to maximise outcomes. This may be of particular importance when we consider that post-chemotherapy liver parenchyma can be challenging to manage intra-operatively and can result in additional blood loss and intra-operative time compared to controls [96].

### 2.5. Future Application of Alternative Biomarkers in the Pre-Operative Setting and LiverMultiScan^TM^

In order to improve patient selection and perioperative planning in liver resection, there is a drive to study factors that can affect liver regeneration, concomitant recovery, and the avoidance of post-hepatectomy liver failure (PHLF). The development of PHLF is closely related to the future liver remnant, and volumetric assays have long been used to predict PHLF risk [98,99,100,101,102,103,104]. However, size alone cannot reliably predict outcomes, especially in the context of underlying liver disease [88,91,105,106,107,108]. It is logical to consider that the function of the liver remnant is also related to postoperative morbidity and mortality, specifically PHLF [109,110,111]. Estimates of hepatic function are based on clinical risk factors, including age, liver disease, and metabolic syndrome [112,113] and specific serological markers of synthetic liver function [114]. Numerous serological markers have been associated with PHLF, but independently, many suffer from low sensitivity and weak positive predictive value [102,114,115,116,117,118,119,120,121,122]. Objective measures of preoperative hepatic function can also be used [123,124,125]. It is well established that steatosis, fibrosis, and cirrhosis play a critical role in liver function, rates of liver regeneration, and PHLF [126]. Furthermore, scores such as APRI/ALBI were shown to detect SOS and CASH [127]. It was also shown to predict outcomes of hepatic resection [128]. Such scores have a valuable role in the selection of patients being worked up for surgical intervention. Furthermore, there is a growing body of evidence suggesting that microRNA (miRNA) signatures may serve as a reliable tool for diagnosis, prognostication, and treatment response biomarkers for various diseases [129,130]. The benefit of miRNAs is that they can be highly specific, cost effective, and easily accessible via biofluids such as blood, urine, and saliva. Specifically in hepatobiliary surgery, miRNA signatures were shown to reliably predict post-operative liver dysfunction, morbidity, and mortality in patients undergoing partial hepatectomy for CRLM and other malignancies [131]. Such miRNA signatures were also shown to outperform indocyanine green (ICG) and volumetric analysis in terms of predicting post-operative liver dysfunction [131]. 

**Figure 2:**
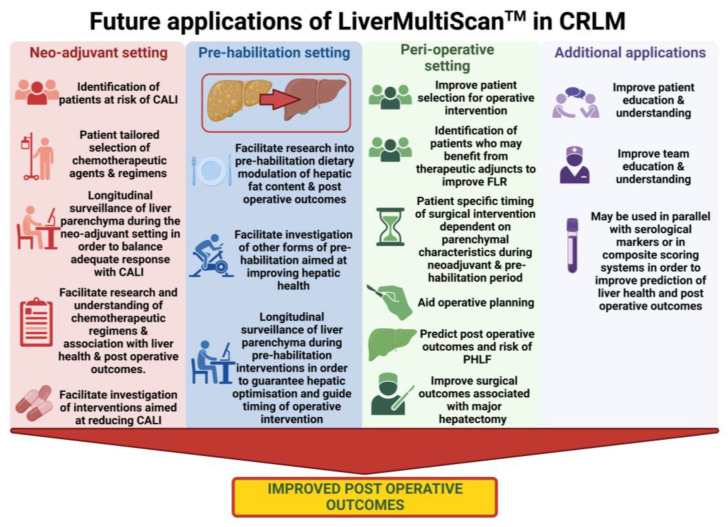
Summary of the potential future applications of the LiverMultiScan^TM^ during the neoadjuvant, pre-habilitation, and peri-operative setting, as well as additional applications throughout the patient journey in order to improve post-operative outcomes. (CRLM = Colorectal Liver Metastasis, CALI = Chemotherapy associated liver injury, FLR = Future liver remnant, PHLF = Post hepatic liver failure).

If LiverMultiScan^TM^ is demonstrated to have clinical applications in patients undergoing chemotherapy and hepatic resections, an important question to address is whether LiverMultiScan^TM^ performs better than serological tests or scores that predict liver health, CALI/CASH/SOS and, importantly, post-operative outcomes such as mortality and PHLF, and whether it can be used in composite with such scores. We foresee non-invasive markers having a pivotal role in the management of CRLM and other primary or secondary liver malignancies. Figure 2 summarises potential applications of the LiverMultiScan^TM^ at various stages in the patient journey.

### 2.6. Pertinent Current Trials Examining the Clinical Applications of LiverMultiScan^TM^ in Liver Surgery for CRLM and Other Hepatic Malignancies

There is currently a trial underway using MR Spectroscopy to longitudinally investigate liver fat content changes during neoadjuvant chemotherapy regimes in CRLM [132]. This trial may shed further light on the issue. Another trial is in the recruitment phase to assess NAFLD after liver transplant using the LiverMultiScan^TM^ (RADICAL2, NCT03165201). One trial has quantified liver health in candidates for hepatic resection using the LiverMultiScan^TM^ (HEPAT1CA, NCT03213314). The same group used cT1 and MRI-PDFF with volume analysis reports for each couinaud segment [133], such that the volume and function of the liver can be assessed together prior to operative intervention. Furthermore, the PRECISION1 trial (NCT04597710) is completed. However, it is not yet published. The results are eagerly anticipated to establish the LiverMultiScans^TM^ clinical value in determining appropriate treatment modalities [134]. This prospective, observational, cohort study aimed to establish the impact of routine use of LiverMultiScan^TM^ data integrated with whole genome sequencing, pathological data, and clinical data on the allocation of treatment options (for example, resection, radiofrequency ablation, venous embolization, chemotherapy, and targeted molecular therapies) in patients with primary or secondary liver cancer. Specific secondary outcome measures, which may shed further light on the role of the LiverMultiScan^TM^ in hepatic surgery, include the correlation of histopathological assessments of liver fat and fibroinflammation with quantitative MRI metrics (i.e cT1 and PDFF), the performance of whole genome sequencing and LiverMultiScan^TM^ for predicting post-surgery length of stay, post-operative liver function, 1 year mortality, and recurrence rates. Following communication with the study group, whilst not yet published, their results suggest the routine use of the LiverMultiScan^TM^ in patients considered for major hepatic resection can encourage alternative therapeutic adjuncts to improve the FLR and prevent PHLF. This group have also demonstrated that LiverMultiScan^TM^ can be used to demonstrate an improvement in the FLR post dual vein embolization in terms of FLR volume, cT1 scores, and PDFF scores in patients with insufficient FLR who required a major hepatectomy [135]. No patient developed PHLF. However, only seven patients underwent dual vein embolization. The group concluded that multiparametric MRI can improve surgical decision making in patients with borderline FLR, preventing PHLF and improving outcomes. Pertinent current and ongoing studies specific to hepatic surgery are detailed in Table 1. 

## 3. Conclusions

Hepatic parenchymal histological markers serve as hallmarks of liver disease, which are likely key determinants for outcomes in the surgical management of malignant hepatic disease. In an era where the options for operative intervention in primary and secondary liver malignancy have seen an explosion of recent major advancements, the number of patients suitable for operative intervention has increased. In doing so, the need to differentiate between those best suited for any given intervention has never been more pertinent. Non-invasive imaging histological markers used as surrogates for liver health and potential for regeneration could pave the way to individualised patient planning approaches, especially if used with current serological and clinical markers of liver function, liver regeneration capacity, and PHLF. Indeed, the LiverMultiScan^TM^ and other non-invasive markers of hepatic parenchyma may be the answer to improving outcomes associated with liver resection. 

Whether it will have any implication on rates of biopsies for liver malignancy is uncertain, as biopsies are rarely carried out. However, its role may come to the forefront of surgical planning, and it will only gain relevance with advancements in technology and expertise. Furthermore, the role of the radiologist in the MDT will only increase with the introduction of novel imaging approaches in order to guide and inform clinicians. This technology still needs to be validated in patient-specific groups (CRLM, other primary and secondary hepatic malignancies, and chemotherapy-associated hepatoxicity), and the real need for it must be clinically translated. Its role will be enlightened by the current studies underway. In future studies, one barrier to consider is a clear understanding of how the performance metrics of the LiverMultiScan^TM^ change over the severity of the CRLM disease burden. Nonetheless, the future looks promising for LiverMultiScan^TM^ and other imaging histological markers.

LiverMultiScan^TM^ is a ‘software as a service’ business model with potential cost implications. Future studies must explore the cost effectiveness and resource impact such a test will have on the oncological and surgical management of liver disease. Important questions to consider are whether it will increase the burden on MRI services and at what financial cost. Local expertise, cost effectiveness, and budgets will likely dictate local access to such investigations in the future. It is also worth noting that the addition of LiverMultiScan^TM^ adds up to 15 min of additional time to a standard liver MRI with contrast, and the implications of this on both services and the patient should be considered. Specifically, some patients may be uncomfortable with small, confined spaces and prolonging this experience may result in claustrophobia. However, in two of our author’s (FW and MR) surgical units, over 200 LiverMultiScan^TM^ MRIs used in combination with Primovist were carried out. All patients were counselled and consented to the slightly longer procedure. There were no incidents of failure to complete the required scan, and no patient volunteered any objections to the experience. Furthermore, there are no additional hospital visits or injections required for LiverMultiScan^TM^. A formal comparative study of patient experience comparing conventional Liver MRI scans with LiverMultiScan^TM^ should be considered. 

Furthermore, with such technology, we are likely to have access to increased information relating to the future liver remnant. In the future, such information may be utilised to identify patients with a severely compromised FLR, which may trigger an alternative therapeutic route to consider in order to optimise the FLR. This may well avoid patients enduring prolonged hospital stays or even mortality as a consequence of PHLF, whilst having positive effects on cost. In fact, in two of our authors’ (FW and MR) affiliated units, the LiverMultiScan^TM^ was used pre-operatively in over 100 consecutive resections. There were no cases of PHLF resulting in delayed discharge. Patients with an abnormal cT1 score, often associated with high-fat content, are diverted to a more parenchymal-sparing procedure; other pre-operative interventions such as dual vein embolization or a two-staged approach with clearance of the left side was initially followed by portal vein ligation and/or portal vein embolization together with hepatic vein embolization. Such findings were formally investigated in a prospective study, which will be published in the near future. If LiverMultiScan^TM^’s role in pre-operative planning for CRLM is validated, its utility will need to be compared to other non-invasive and potentially more cost-effective serological tests/scores. 

We would encourage large volume centres to collaborate in future trials to strengthen the power of studies and, in doing so, provide valuable insights into the predictive value of such non-invasive tests in morbidity and mortality post hepatectomy, as well as the exact role grades of histological changes in the liver play in outcomes. Such studies should investigate non-invasive imaging markers and serological markers (such as APRI/ALBI) in parallel. Indeed, the LiverMultiScan^TM^ and other imaging markers may complement other assays of liver health in order to minimise the risk associated with extended hepatectomy. It is an exciting prospect that with such markers of liver parenchyma status, we are likely to see the advent of novel composite scoring systems, which will use this information in combination with other serological and imaging assays to predict outcomes of surgery and optimise patient and therapeutic selection and timing. In doing so, it could provide real-time information, which may guide patient-tailored decision making regarding the ideal timing for surgical intervention during the neoadjuvant period. Robust clinical trials are warranted to validate such tools. 

## Figures and Tables

**Table 1:** Pertinent current and future studies investigating the role of LiverMultiScan in Hepatic surgery for CRLM.

Current Studies						
Reference	Title	Study Design	Cohort and Study Information	Findings	Status	Possible Future Applications
Mole et al. 2020 [12]	HepaT1ca (NCT03213314)	An observational clinical cohort study in two tertiary referral HPB centres.	Included 149 participants. Combined 3D volumetric assessment of FLR with cT1 score prior to treatment. Total of 135 participants underwent liver resection. Majority of participants had CRLM (*n* = 114). The remaining had HCC (*n* = 6), CCA (*n* = 1) or other secondary malignancies (*n* = 14). Imaging biomarkers (cT1 and PDFF) correlated with histological assessment from intra-operative tissue samples.	− cT1 correlated with histological scoring of ballooning and inflammation. PDFF correlated with steatosis scoring. − Elevated pre-operative cT1 score correlated with prolonged hospital stay vs. normal pre-operative cT1 score (6.5 days vs. 5 days, *p* = 0.005). − A composite score combining FLR and cT1 predicted poor liver performance (as measured by a modified Hyder–Pawlik score) on post-operative day 5 (AUROC = 0.78). − The same composite score correlated with liver regeneration at 3 months post resection.	Completed and published study	Correlates with histological assays of fibroinflammation and steatosis and may circumvent need for a pre-operative biopsy in select patients in the pre-operative setting. Abnormal cT1 score can help Identify patients at risk of prolonged hospital stay and poor liver performance post operatively. Informed risk stratification of patients and personalised pre-operative decision making. Potential value of composite scoring systems in predicting post-operative outcomes and liver regeneration capacity.
Sethi et al. 2021 [43]	Quantitative multiparametric MRI allows safe surgical planning in patients undergoing liver resection for colorectal liver metastases: report of two patients	Retrospective case presentation of 2 patients included in the observational clinical trial, HepaT1ca (NCT03213314)	Both patients had CRLM and underwent extended right hepatectomy with estimated FLR 30%. Comparable pre-operative characteristics in terms of demographics, imaging, and baseline laboratory values.	− Patient 1 developed PHLF and prolonged admission. Patient 2 had an uneventful post operative clinical course. − Retrospective evaluation of multi-parametric MRI using LiverMultiScan^TM^ showed Patient 1 had elevated fibro-inflammatory disease (cT1 = 829 ms) and steatosis (PDFF = 14%). Patient 2 had normal parametres (cT1 = 745 ms and PDFF = 2.4%).	Completed and published study	Potential objective evaluation of liver parenchyma, which can reveal significant underlying liver disease. This may aid/change decision making regarding pre-operative optimisation of the FLR in order to improve post-operative outcomes.
McKay et al. 2021 [33]	Patient understanding and experience of non-invasive imaging diagnostic techniques and the liver patient pathway	Cross-sectional study. Pre- and post- LiverMultiScan self-rated questionnaire on understanding of liver health. Post- LiverMultiScan semi-structured qualitative interview re. patient experience, understanding of the report and how to improve experience and delivery of information.	101 participants included with a spectrum of liver disease diagnosis, including cancer.	− Self-reported understanding of liver health increased significantly from 6.28 to 9.22 (+2.94) − Analysis of semi-structured interviews revealed that (1) The presentation and discussion of the LiverMultiScan visual report was an effective contributor to better patient understanding. (2) Patients demonstrated preference for non-invasive tests over biopsies. (3) Patients reported positive experiences with the LiverMultiScan.	Completed and published study	Visual reports of liver health may increase patient understanding of their disease care and overall experience. Potential for improving patient engagement with care.
Sundaravadanan et al. 2022 [135]	Multimetric MRI detects improved quality of the future liver remnant post-dual vein embolization—a novel finding.	Presentation abstract	Analysis of 81 patients with CRLM considered for liver resection, recruited in Precision1 trial (NCT04597710). Seven patients with CRLM had multiparametric MRI (including LiverMultiScan and volumetric assay) pre- and post- DVE.	− DVE resulted in significant FLR volume increase, as well as reduction in FLR cT1 scores; median 747.33 ms (range 684–884 ms) from median 771.25 ms (range 726–945 ms), *p* = 0.047. Median PDFF scores also improved post DVE. − No patient developed PHLF.	Presented with published abstract	Demonstrating potential role in clinical trials for interventions aimed at optimising FLR. Aids in surgical decision making in patients with borderline FLR in order to optimise FLR and improve outcomes.
Welsh et al. 2023 [136]	Quantitative liver health imaging impacts surgical decision making and improves clinical outcomes in colorectal liver metastasis surgery	Comparative observational cohort study, including prospective cohort from Precision1 trial vs. analysis of a historical similar cohort	Analysis of the clinical utility of mpMRI in 81 patients with CRLM considered for liver resection (recruited in the Precision1 trial, NCT04597710). Clinical utility as measured by a change in the surgical pan. Post operative clinical outcomes of the cohort were compared with a similar historical cohort including 97 patients with CRLM, as well as other hepatic cancers. Both cohorts underwent mpMRI, including cT1, T2, and PDFF. However, information obtained from mpMRI was not used to alter surgical plans in the comparator cohort.	− Examination of mpMRI reports resulted in a change in surgical plan in 29/81 cases in the Precision1 cohort, whether that be a more aggressive or conservative resection, dietary modification or a two staged/DVE approach. − Mean length of stay in the comparator dataset was 6.7 days (±9.1) vs. 5.3 (±2.1) (*p* = 0.147). Notably, protracted length of stay (>14 days) was greater in the comparator dataset, 5% vs. 1% (*p* = 0.136). − Another pertinent finding in this study; poor liver health was underestimated in up to 40% of patients planned for liver resection.	Preprint article awaiting peer review	mpMRI utilising LiverMultiScan in pre-operative planning may improve LoS. mpMRI may alter surgical strategy or provide confidence with the proposed treatment strategy. mpMRI may pick up underestimated liver health using conventional assays of liver health and volume.
Future studies						
Reference	Title	Aim	Study design	Primary objective/end points	Secondary objective/end points	
Welsh et al. 2022 [134]	Precision1 Trial: Precision medicine for liver tumours with quantitative MRI and whole genome sequencing. NCT04597710.	Whole genome sequencing (WGS) integration with quantitative MRI and histopathology data to produce a software product to inform management of patients with liver tumours.	A single centre prospective observational cohort study of up to 200 adult participants being considered for liver resection of a primary or secondary liver cancer.	To determine the utility of WGS to aid clinical decision making in patients referred for liver resection. Evaluated retrospectively, with clinically actionable data defined as data resulting in clinicians choosing a different medical intervention to the current standard of care.	To determine the utility of LiverMultiScan to aid clinical decision making in patients referred for liver resection. Evaluated retrospectively as proportion of patients for whom clinically actionable data are provided by LiverMultiScan. To compare computationally derived pathology results with human pathologist assessments. To compare histopathological assessment of liver fat and fibro-inflammation with LiverMultiScan (cT1 and PDFF). To evaluate long term outcomes and recurrence rates/patterns of patients as it relates to WGS and imaging. To evaluate if WGS enables better stratification of patients pre-operatively.	
Parmar et al. 2023 [132]	CoNoR Study: A prospective multi-step study of the potential added benefit of two novel assessment tools in colorectal liver metastases technical resectability decision-making	To evaluate the potential added value of two novel assessment tools (Hepatica, i.e., LiverMultiScan with 3D volumetric assay, and LiMax) in CRLM resectability decision making	A multistep systematic approach of systematic review, international expert interviews, international questionnaire and internation case-based surveys. Including international HPB senior community.	The added value of Hepatica and LiMAx in CRLM technical resectability decision making, assessed by measuring the following in HPB experts: Proportion of change in resectability decision making resulting from the novel tests Proportion of change in planned operative strategy resulting from novel tests.	1. Variability in CRLM resectability decision making. 2. Opinions on the role of novel tools in CRLM resectability decision making	
